# Characteristic Face: A Key Indicator for Direct Diagnosis of 22q11.2 Deletions in Chinese Velocardiofacial Syndrome Patients

**DOI:** 10.1371/journal.pone.0054404

**Published:** 2013-01-16

**Authors:** Dandan Wu, Yang Chen, Chen Xu, Ke Wang, Huijun Wang, Fengyun Zheng, Duan Ma, Guomin Wang

**Affiliations:** 1 Department of Oral & Cranio-maxillofacial Science, Shanghai 9th People’s Hospital, College of Stomatology, School of Medicine, Shanghai Jiao Tong University, Shanghai Key Laboratory of Stomatology, Shanghai, P. R. China; 2 Department of Oral and Maxillofacial Surgery, The Affiliated Hospital, Medical School, Qingdao University, Qingdao, P. R. China; 3 Children’s Hospital, Fudan University, Shanghai, P. R. China; 4 Key Laboratory of Molecular Medicine, Ministry of Education, Department of Biochemistry and Molecular Biology, Institute of Medical Sciences, Shanghai Medical College, Fudan University, Shanghai, P. R. China; Pasteur Institute of Lille, France

## Abstract

Velocardiofacial syndrome (VCFS) is a disease in human with an expansive phenotypic spectrum and diverse genetic mechanisms mainly associated with copy number variations (CNVs) on 22q11.2 or other chromosomes. However, the correlations between CNVs and phenotypes remain ambiguous. This study aims to analyze the types and sizes of CNVs in VCFS patients, to define whether correlations exist between CNVs and clinical manifestations in Chinese VCFS patients. In total, 55 clinically suspected Chinese VCFS patients and 100 normal controls were detected by multiplex ligation-dependent probe amplification (MLPA). The data from MLPA and all the detailed clinical features of the objects were documented and analyzed. A total of 44 patients (80.0%) were diagnosed with CNVs on 22q11.2. Among them, 43 (78.2%) presented with 22q11.2 heterozygous deletions, of whom 40 (93.0%) had typical 3-Mb deletion, and 3 (7.0%) exhibited proximal 1.5-Mb deletion; no patient was found with atypical deletion on 22q11.2. One patient (1.8%) presented with a 3-Mb duplication mapping to the typical 3-Mb region on 22q11.2, while none of the chromosomal abnormalities in the MLPA kit were found in the other 11 patients and 100 normal controls. All the 43 patients with 22q11.2 deletions displayed characteristic face and palatal anomalies; 37 of them (86.0%) had cognitive or behavioral disorders, and 23 (53.5%) suffered from immune deficiencies; 10 patients (23.3%) manifested congenital heart diseases. Interestingly, all patients with the characteristic face had 22q11.2 heterozygous deletions, but no difference in phenotypic spectrum was observed between 3-Mb and 1.5-Mb deletions. Our data suggest that the characteristic face can be used as a key indicator for direct diagnosis of 22q11.2 deletions in Chinese VCFS patients.

## Introduction

VCFS, also known as DiGeorge syndrome, conotruncal anomalies face syndrome, and CATCH 22, is firstly termed by *Shprintzen et al.* in 1978 and exhibits an expansive phenotype with more than 180 clinical features involving almost every organ and system [1], [2]. The major symptoms include congenital heart disease, particular conotruncal malformation, characteristic face, palatal abnormality, immune deficiency, and cognitive or behavioral disorder. Its minor features involve growth retardation, neonatal hypocalcemia, feeding difficulty, hearing loss, limb deformity, and so on. The penetrance of each clinical feature is different; no single phenotype occurs in all patients, and none is obligatory [3].

VCFS is also one of the most common human genomic disorders with a population prevalence ranging from approximately 1∶2000 to 1∶7000 [4]. Only about 5–15% patients inherit the disease from parents, while most of them are sporadic (*de novo*) due to a heterozygous deletion caused by nonallelic homologous recombination (NAHR), mainly mediated by the low copy repeats (LCRs) on 22q11.2. Three types of deletion on 22q11.2 have been reported so far. While most the cases have typical 3-Mb deletion with breakpoints flanked by LCR A and D, some cases carry proximal 1.5-Mb deletion flanked by LCR A and B within the typically deleted region (TDR); only a few cases show atypical deletions overlapping or nonoverlapping with the TDR [5]. Researchers also found that certain cases with 22q11.2 duplication or CNVs on other chromosomes, such as 4q, 8p, 10p, and 17p, presented with clinical phenotypes similar to those of VCFS; these cases were also defined as VCFS by some geneticists and clinicians, who believed that such cases increased the diversity of genetic mechanism of VCFS [6]–[10].

Although recent studies have improved our understanding of the pathogenesis of VCFS, the correlations between the phenotypes and genotypes of CNVs remain ambiguous [11]. While discordant types of CNVs can occur in the cases with identical phenotypes, concordant chromosomal aberrations may result in variable expressions, even within families or between homozygotic twins [12], [13]. Thus, exactly determining the specified clinical features for each type of CNVs will facilitate genetic counseling and health care.

This study has detected 55 clinically-suspected Chinese VCFS patients by MLPA, with 100 healthy subjects as control, aiming to reveal the types and sizes of CNVs in VCFS patients with different phenotypes and explore the correlations between CNVs and clinical findings in Chinese VCFS patients.

## Methods

### Patients

**Figure 1:**
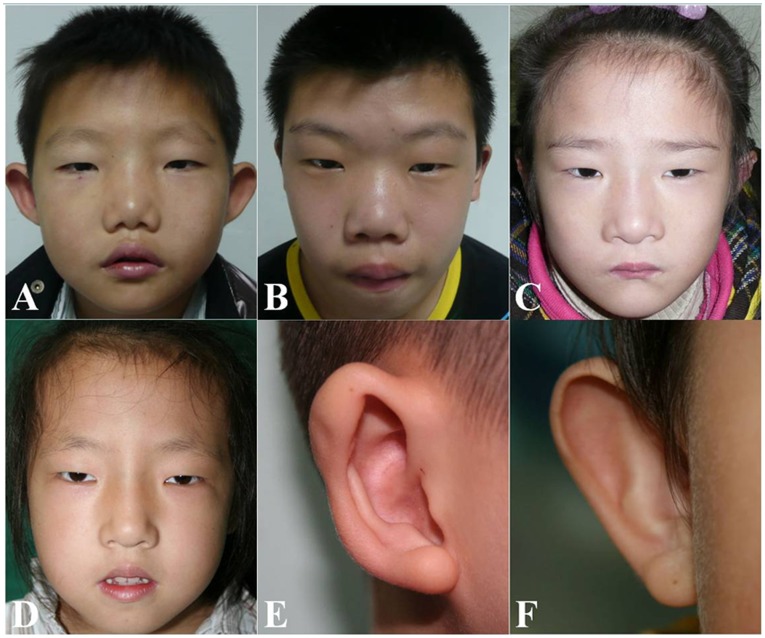
Characteristic face of VCFS. These Chinese VCFS patients all presented with a characteristic face, consisting of vertically long face, narrow palpebral fissures, fleshy nose with a broad nasal root, flattened malar region, retrognathia, and sometimes overfolded helix (E) or cup-shaped ear (F).

All 55 patients enrolled in this study were from the Center for Cleft Lip and Palate, Shanghai Ninth People’s Hospital, Shanghai Jiao Tong University School of Medicine, and all the clinical findings in these patients were confirmed with physical or auxiliary examinations by specialists. Patients from No. 1–43 commonly presented with characteristic face and palatal abnormality, and most of them displayed other features such as congenital heart diseases, immune deficiency, and cognitive or behavioral disorders. Patients No. 44 presented with palatal abnormality, immune deficiency, mild mental retardation, low set ears, and conductive hearing disturbance. Patients No. 45–54 commonly presented with congenital heart diseases and palatal abnormality, and some of them showed other features too. Patients No. 55 presented with palatal abnormality, mild mental retardation, and conductive hearing disturbance (Table 1). The identified congenital heart diseases included tetralogy of Fallot, interrupted aortic arch, ventricular septal defect, atrial septal defect, and patent ductus arteriosus. The characteristic face consisted of vertically long face, narrow palpebral fissures, fleshy nose with a broad nasal root, flattened malar region, and retrognathia (Figure 1). The palatal abnormalities involved congenital velopharyngeal insufficiency, submucosal cleft palate, occult submucous cleft palate, and overt cleft palate. Patients who had thymic hypoplasia, T-cell deficiency, or history of recurrent infections, were diagnosed with immune deficiency. All the cognitive or behavioral disorders in VCFS patients were mild.

**Table 1:** Summary of patient data.

Case no.	Sex	Age (years)	Phenotypes						Molecular diagnosis
			CHD	Characteristic face	Palatal anomaly	Immune deficiency	Cognitive/behavioral disorder	Other features	
1	M	5	+	+	+	+	+	Epilepsy	3Mb 22q11del
2	F	5	–	+	+	–	+	–	3Mb 22q11del
3	F	10	–	+	+	+	+	–	3Mb 22q11del
4	M	6	–	+	+	+	–	Hypocalcemia	3Mb 22q11del
5	F	8	–	+	+	+	+	Epilepsy	3Mb 22q11del
6	F	12	–	+	+	–	+	Low set ears	3Mb 22q11del
7	F	21	–	+	+	+	+	–	3Mb 22q11del
8	F	6	–	+	+	+	+	–	3Mb 22q11del
9	M	5	–	+	+	–	+	Cup-shaped ears, anal fistula	3Mb 22q11del
10	F	4	–	+	+	+	+	Cup-shaped ears, hypospadias	3Mb 22q11del
11	M	6	–	+	+	+	–	–	3Mb 22q11del
12	M	13	–	+	+	+	+	Cup-shaped ears	3Mb 22q11del
13	M	12	+	+	+	–	+	Hypocalcification of teeth	3Mb 22q11del
14	F	13	–	+	+	–	+	–	3Mb 22q11del
15	F	11	–	+	+	–	+	–	3Mb 22q11del
16	F	10	–	+	+	+	+	Brachydactyly	3Mb 22q11del
17	M	8	+	+	+	+	+	–	3Mb 22q11del
18	M	6	+	+	+	+	+	–	3Mb 22q11del
19	F	3	–	+	+	–	+	Growth retardation, overfolded helix, oblique inguinal hernia	3Mb 22q11del
20	M	13	+	+	+	–	+	–	3Mb 22q11del
21	M	2	+	+	+	+	+	Overfolded helix, oblique inguinal hernia	3Mb 22q11del
22	M	7	–	+	+	–	+	Cup-shaped ears	3Mb 22q11del
23	F	11	+	+	+	–	+	Cup-shaped ears	3Mb 22q11del
24	F	6	–	+	+	+	+	Overfolded helix	3Mb 22q11del
25	M	20	–	+	+	+	+	Epilepsy	3Mb 22q11del
26	M	2	+	+	+	–	–	Epilepsy, hypomyotonia	3Mb 22q11del
27	M	8	–	+	+	+	+	Growth retardation	3Mb 22q11del
28	F	4	–	+	+	+	+	Growth retardation, cup-shaped ears	3Mb 22q11del
29	M	18	–	+	+	–	+	Overfolded helix	3Mb 22q11del
30	F	5	–	+	+	+	–	Cup-shaped ears	3Mb 22q11del
31	M	3	–	+	+	+	–	Cup-shaped ears	3Mb 22q11del
32	M	14	–	+	+	–	+	Low set and cup-shaped ears	3Mb 22q11del
33	M	13	–	+	+	–	+	Overfolded helix	3Mb 22q11del
34	F	10	–	+	+	–	+	Overfolded helix	3Mb 22q11del
35	M	15	–	+	+	–	+	–	3Mb 22q11del
36	F	5	–	+	+	–	–	Cup-shaped ears	3Mb 22q11del
37	F	12	–	+	+	+	+	Epilepsy	3Mb 22q11del
38	M	11	–	+	+	+	+	Small stature, low set ears, congenital odontosteresis, digital hyperextensibility, cryptorchidism	3Mb 22q11del
39	F	13	+	+	+	+	+	–	3Mb 22q11del
40	M	4	+	+	+	+	+	Overfolded helix	3Mb 22q11del
41	M	17	–	+	+	–	+	Small stature, cup-shaped ears, oblique inguinal hernia	1.5Mb 22q11del
42	F	18	–	+	+	–	+	–	1.5Mb 22q11del
43	F	12	–	+	+	–	+	Cup-shaped ears	1.5Mb 22q11del
44	M	4	–	–	+	+	+	Low set ears, conductive hearing disturbance	3Mb 22q11dup
45	F	2	+	–	+	–	–	Low set ears	–
46	F	2	+	–	+	–	–	Exotropia, cup-shaped ears, syndactyly	–
47	M	7	+	–	+	–	+	Exotropia, cleft lip, preaxial polydactyly, oblique inguinal hernia, bifurcation of the left third rib, growth retardation	–
48	F	2	+	–	+	–	–	–	–
49	M	2	+	–	+	–	–	–	–
50	F	2	+	–	+	–	–	Growth retardation	–
51	M	24	+	–	+	–	–	Cleft lip	–
52	F	21	+	–	+	–	–	Growth retardation, partial sternal absence, bifurcation of the right fourth rib	–
53	F	3	+	–	+	–	–	Cleft lip	–
54	F	2	+	–	+	–	–	–	–
55	M	5	–	–	+	–	+	Conductive hearing disturbance	–

M, male; F, female; CHD, congenital heart disease; +, presence of symptoms; −, absence of symptoms or no CVNs in MLPA detection; del, deletion; dup, duplication.

The present study was carried out according to the principles of the Declaration of Helsinki and approved by the Shanghai Ninth People’s Hospital Ethics Committee. Written informed consents were obtained from all participants, and written permission to use the images in this study was also obtained from the participated patients or their parents.

### DNA Extraction

Genomic DNA was extracted from the whole peripheral blood using QIAGEN kit (QIAGEN, Hilden, Germany) according to the manufacturer’s instructions, and a concentration of 10–50 ng/uL DNA was suitable for MLPA detecting.

### MLPA Analysis

The MLPA detection was performed using SALSA MLPA KIT P250-B1 DiGeorge (MRC-Holland, Amsterdam, Netherlands) according to the provider’s protocol. Amplification products were detected and quantified by capillary electrophoresis on an ABI 3130XL Genetic Analyser (Applied Biosystems, Foster City, CA). Finally, the files of electropherograms were imported into and analyzed in GeneMarker software V1.8 (Softgenetics, State College, PA).

The MLPA kit used in this study contained 48 probes for 48 different genes. There were 29 probes targeting 22q11 region, among which 9 probes were between LCR A and B; 3 probes between LCR B and C; 2 probes between LCR C and D; 3 probes between LCR D and E; 4 probes between LCR E and F; 2 probes between LCR F and G; 1 probe between LCR C and D; and 5 probes in Cat Eye syndrome region proximal to LCR A. The other 19 probes located in other locus outside 22q11. Among them, 2 probes were on 4q34-qter; 3 probes on 8p23; 2 probes on 9q34.3; 6 probes on 10p14; 4 probes on17p13.3; and 2 probes on 22q13.

Each sample was detected in triplicate by MLPA, and the replicates were detected on different days.

## Results

**Figure 2:**
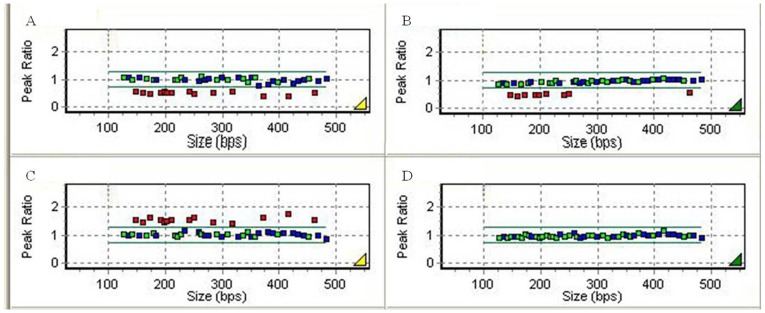
Data of MLPA analysis with P250-B1 DiGeorge kit. The four graphs represent four patients’ data analyzed by MLPA. In each graph, the spots represent MLPA probes, the upper green line indicates a peak ratio of 1.3 and any probes above this line represent a duplication, the lower green line indicates a peak ratio of 0.75 and any probes below this line represents a deletion, and the probes between the two lines are considered as normal two copies. (**A**): A patient with 22q11.2 deletion spanning 3Mb TDR (red spots). (**B**) A patient with 22q11.2 deletion spanning proximal 1.5Mb (red spots) within TDR. (**C**): A patient with 22q11.2 duplication (red spots) mapping to 3Mb TDR. (**D**) A patient with normal copy probes.

**Figure 3:**
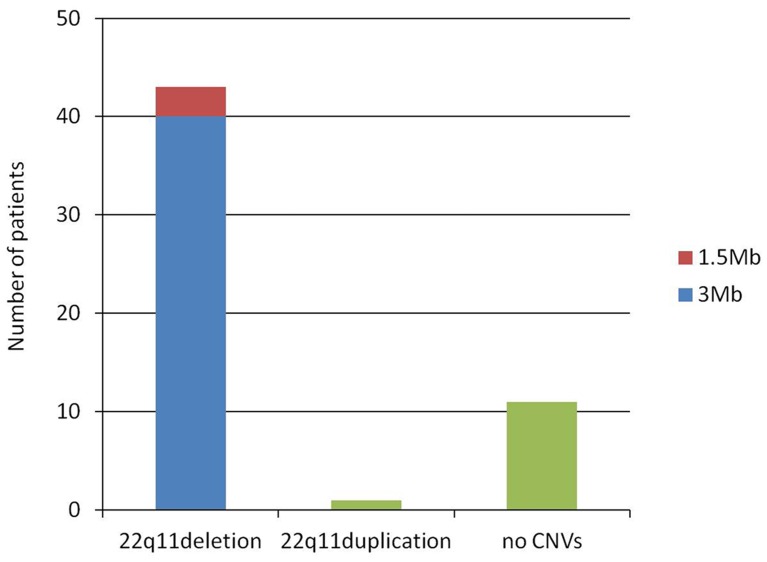
The results of 55 patients detected by MLPA. A total of 43 cases (78.2%) showed 22q11.2 heterozygous deletion, of whom 40 (93.0%) exhibited typical 3-Mb deletion, while 3 (7.0%) showed proximal 1.5-Mb deletion; no case was found having atypical deletion on 22q11.2. Only 1 case (1.8%) had 3-Mb duplication. None of the chromosomal abnormalities in the MLPA kit were found in the other 11 patients.

Of the 55 patients, 44 (80.0%) were confirmed with CNVs on 22q11.2 by MLPA analysis. Among them, 43 cases (78.2%) showed 22q11.2 heterozygous deletion, of whom 40 (93.0%) exhibited typical 3-Mb deletion with breakpoints between LCR A and D, while 3 (7.0%) displayed proximal 1.5-Mb deletion between LCR A and B; no case was found with atypical deletion on 22q11.2. Additionally, 1 case (1.8%) had 3-Mb duplication mapping to the typical 3-Mb region on 22q11.2 (Figure 2 and 3). None of the chromosomal abnormalities in the MLPA kit were detected in the rest 11 patients and 100 normal controls.

**Figure 4:**
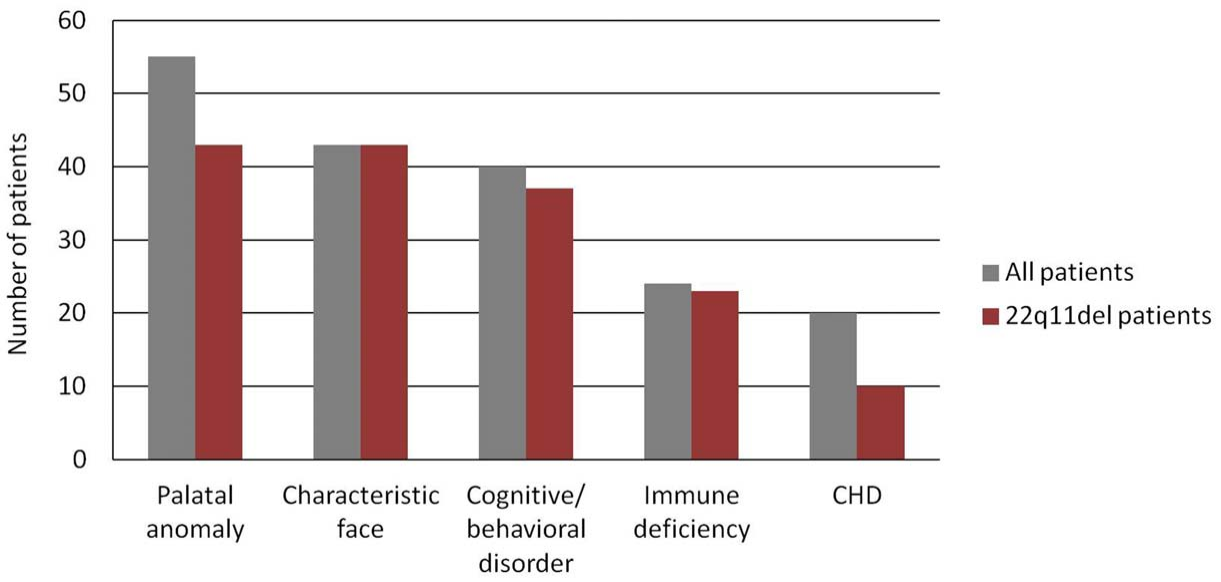
Number of patients presenting with major clinical features of VCFS. All cases with characteristic faces exhibited 22q11.2 heterozygous deletions.

According to the clinical features, all 55 VCFS patients (100%) had palatal anomalies; 43 patients (78.2%) exhibited characteristic face; 40 patients (72.7%) displayed cognitive or behavioral disorders; 24 patients (43.6%) had immune deficiency; and 20 patients (36.4%) suffered from congenital heart diseases. Other features with lower frequencies were ear deformities, conductive hearing disturbance, exotropia, cleft lip, tooth hypocalcification, congenital odontosteresis, brachydactyly, syndactyly, preaxial polydactyly, digital hyperextensibility, epilepsy, hypocalcemia, growth retardation, small stature, partial sternal absence, bifid rib, cryptorchidism, hypospadias, oblique inguinal hernia, anal fistula, and hypomyotonia (Table 1). Of the 43 patients with 22q11.2del (No. 1–43), all (100%) exhibited characteristic face and palatal anomalies; 37 patients (86.0%) showed cognitive or behavioral disorders; 23 patients (53.5%) had immune deficiency; and 10 patients (23.3%) suffered from congenital heart diseases (Figure 4). The patient with 22q11dup (No. 44) displayed palatal abnormality, immune deficiency, mild mental retardation, low set ears, and conductive hearing disturbance.

Comparing the clinical findings and molecular diagnosis, we found that all the cases with characteristic faces harbored 22q11.2 heterozygous deletions, but there was no difference in phenotypic spectrum between the 3-Mb and 1.5-Mb deletions.

## Discussion

As a multiple anomaly syndrome, VCFS’s phenotypes are complex and diverse with expression variable in each patient. There are no definitive or minimal diagnostic criteria for this disorder, and most of the clinical findings are easy to overlap with other genetic disorders such as, Noonan syndrome, Alagille syndrome, Goldenhar syndrome, and Smith-Lemli-Opitz syndrome, as well as CHARGE and VATER associations [3]. Therefore, the clinical diagnosis of VCFS largely depends on clinicians’ experience and hence is often biased or wrong. However, accurate diagnosis of this disorder is crucial for treatment [14]. Some features not presenting at birth may have a late onset; thus, an early identification can lead to more effective therapeutic regimen with better prognosis [15].

Molecular diagnosis is considered as a more advanced and accurate method for defining VCFS [16]. Studies showed that more than 90% VCFS cases have microdeletion on 22q11.2, among which 85–90% were typical 3-Mb deletion; 10–12% proximal 1.5-Mb deletion, and only a few were atypical deletions [5]. Moreover, a small number of VCFS-like cases harboring a microduplication on 22q11.2 or CNVs on other chromosomes (4q, 8p, 10p, and 17p), such as partial monosomies and trisomies, have also been reported as etiologic heterogeneity that causes clinical variability [17]. Identification of these variant cases is of particular interest, because it may provide insight into the genes or genomic regions, which are crucial for specific phenotypic manifestations, and may help to elucidate the mechanisms of deletion and duplication [18]. Oh *et al.* analyzed phenotypes of 16 patients with 22q11.2 deletions and revealed the correlation of characteristic face and small stature with 22q11.2 deletions [19]. Rauch *et al.* stated that atypical clinical findings related to atypical 22q11.2 deletions, and that the distally nested interval of TDR containing the gene of *CRKL* contributed to major mental impairment [20]. Yatsenko *et al.* found that atrial septal defect might be the most common cardiac anomaly associated with haploinsufficiency of genes on 10p, a commonly accepted second critical region for DiGeorge syndrome [21]. However, most of the investigators failed to define any correlations between molecular diagnosis and clinical findings.

This study mainly has analyzed the major compositions of phenotypes and types of associated chromosomal aberrations and identified the correlations between CNVs and clinical features in Chinese VCFS patients. Our data indicated that 43 cases (78.2%) among all patients suspected for VCFS presented with 22q11.2 heterozygous deletions; of them, 40 (93.0%) exhibited typical 3-Mb deletion, and 3 (7.0%) displayed proximal 1.5-Mb deletion. In theory, both deletion and duplication events should occur in equal proportions, as a result of NAHR due to unequal crossovers of LCRs [22]. However, the reported frequency of 22q11.2 duplications was much lower than that of 22q11.2 deletions [23]–[25]. In consistence, only 1 case (1.8%) in this study showed 3-Mb duplication mapping to the TDR on 22q11.2.

In terms of phenotype, all the 43 patients (100%) with 22q11.2del displayed the characteristic face and palatal abnormalities, indicating that both features, the characteristic face and palatal abnormalities, may strongly associate with the 22q11.2 deletions in Chinese patients. The frequencies of cognitive/behavioral disorders and immune deficiency were 86.0% and 53.5% respectively, similar to those of recent reports [26], [27], suggesting that these are also common features demand more attentions during the patients’ growth and development. Surprisingly, congenital heart diseases, the major symptoms of 22q11.2del according to some scholars [4], [5], were observed in only 23.3% cases in this study. Variable penetrance in different races or bias in patient selection may be responsible for this phenomenon. Some investigators suggested that the absence of cardiac anomalies should not impede the clinical diagnosis for a patient with VCFS [28], [29]. In addition, the clinical manifestations of thepatient with 22q11dup seem milder than those of patients with 22q11del herein. Most importantly, our data indicate that all cases with the characteristic face exhibited 22q11.2 heterozygous 3-Mb or 1.5-Mb deletion, suggesting that characteristic face that commonly presents in the majority of Caucasian individuals [30] forms an indicative factor for direct diagnosis of 22q11.2 deletions in Chinese VCFS patients. There seems to be a discrepancy between the present study and the study of Xu et al. [31], in which some of the 13 Chinese 22q11del patients did not show a characteristic face. This may be explained by that the patients in their study were mostly infants, whose facial characteristics might not have been yet recognizable, i.e., the phenotypes were not manifested yet. The phenotypic spectrums between 3-Mb and 1.5-Mb deletions show no difference in this study, which is consistent with previous reports [4], [32]. The proximal 1.5-Mb region, nested in 3-Mb TDR, is considered as the critical region for the syndrome; many genes in this region have been tested for causative effects, especially *TBX1*, which has been defined as a major candidate gene for this disorder [16]. However, whether *TBX1* mutations in human cause VCFS remains controversial. Only 9 patients with VCFS have been identified to have *TBX1* mutations [33]–[35], while a large number of patients were determined to be negative for *TBX1* mutations [36], [37], and it seems that this disease was not caused by a single gene mutation or dosage alteration, but resulted from a combined effect of many genes [38].

In comparison to the standard fluorescence *in situ* hybridization (FISH), MLPA used in this study has been recognized as a more rapid, highly cost-effective, and sensitive method for detecting various types of CNVs, especially in the 22q11.2 region [39]. It not only can identify certain atypical aberrations on 22q11.2 that the commercially available FISH probe of TUPLE1 or N25 is unable to detect, but also can provide the sizes of these aberrations simultaneously [40]. The MLPA of P250-B1 DiGeorge kit containing probes targeting 22q11 and other above described VCFS-associated chromosomes may serve as a more reliable tool for the molecular diagnosis of VCFS.

Nonetheless, the present study has limitations. Firstly, our results were obtained by analyzing 55 suspected VCFS cases; further studies with a larger sample size may provide more detailed and accurate data. Secondly, since all the cases were from one center that mainly engages in craniofacial researches, selection bias may exist; future multicenter researches by multidisciplinary teams may avoid the possible inclusion bias. Thirdly, the 11 cases without CNVs may relate to other chromosomal aberrations out of MLPA locus, suggesting that more genetic approaches should be used in future to grasp larger sets of data. Overcoming these limitations will improve our ability to determine the types of CNVs contributing to specified abnormal phenotypes, and to eventually facilitate a more consistent application of these techniques in genetic counseling.

### Conclusions

VCFS presents with variable phenotypes and diverse chromosomal aberrations. Accurate diagnosis of VCFS not only benefits the genetic counseling and patient care, but also helps patients to obtain effective therapeutic regimen and good prognosis. This study has analyzed the clinical phenotypes and molecular diagnosis of 55 Chinese VCFS patients and revealed that all the cases with characteristic face exhibited 22q11.2 heterozygous 3-Mb or 1.5-Mb deletion, indicating that the characteristic face can be used as a key indicator for direct diagnosis of VCFS with 22q11.2 deletions in Chinese patients.
